# “Sweet Flavonoids”: Glycosidase-Catalyzed Modifications

**DOI:** 10.3390/ijms19072126

**Published:** 2018-07-21

**Authors:** Kristýna Slámová, Jana Kapešová, Kateřina Valentová

**Affiliations:** Laboratory of Biotransformation, Institute of Microbiology, Czech Academy of Sciences, Vídeňská 1083, CZ-14220 Prague 4, Czech Republic; slamova.kristyna@gmail.com (K.S.); janakapesova@gmail.com (J.K.)

**Keywords:** enzymatic hydrolysis, quercetin, hesperetin, naringenin, rutinosidase, rhamnosidase, puerarin, catechin, icariin, transglycosylation

## Abstract

Natural flavonoids, especially in their glycosylated forms, are the most abundant phenolic compounds found in plants, fruit, and vegetables. They exhibit a large variety of beneficial physiological effects, which makes them generally interesting in a broad spectrum of scientific areas. In this review, we focus on recent advances in the modifications of the glycosidic parts of various flavonoids employing glycosidases, covering both selective trimming of the sugar moieties and glycosylation of flavonoid aglycones by natural and mutant glycosidases. Glycosylation of flavonoids strongly enhances their water solubility and thus increases their bioavailability. Antioxidant and most biological activities are usually less pronounced in glycosides, but some specific bioactivities are enhanced. The presence of l-rhamnose (6-deoxy-α-l-mannopyranose) in rhamnosides, rutinosides (rutin, hesperidin) and neohesperidosides (naringin) plays an important role in properties of flavonoid glycosides, which can be considered as “pro-drugs”. The natural hydrolytic activity of glycosidases is widely employed in biotechnological deglycosylation processes producing respective aglycones or partially deglycosylated flavonoids. Moreover, deglycosylation is quite commonly used in the food industry aiming at the improvement of sensoric properties of beverages such as debittering of citrus juices or enhancement of wine aromas. Therefore, natural and mutant glycosidases are excellent tools for modifications of flavonoid glycosides.

## 1. Introduction

**Figure 1:**
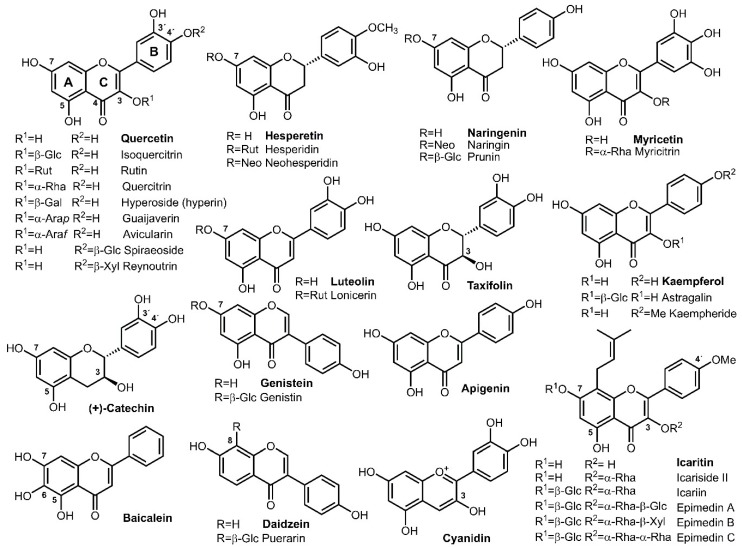
Structures of selected biotechnologically valuable flavonoids (in bold) and their glycosides. Glc: d-glucopyranosyl, Rha: l-rhamnopyranosyl (6-deoxy-l-mannopyranosyl), Gal: d-galactopyranosyl, Ara*p*: l-arabinopyranosyl, Ara*f*: l-arabinofuranosyl, Xyl: d-xylopyranosyl, Rut: rutinosyl (6-*O*-α-l-rhamnopyranosyl-β-d-glucopyranosyl), Neo: neohesperidosyl (2-*O*-α-l-rhamnopyranosyl-d-glucopyranosyl), α-Rha-β-Glc: 2-*O*-β-d-glucopyranosyl-α-l-rhamnopyranosyl, α-Rha-β-Xyl: 2-*O*-β-d-xylopyranosyl-α-l-rhamnopyranosyl, α-Rha-α-Rha: 2-*O*-α-l-rhamnopyranosyl-α-l-rhamnopyranosyl.

Flavonoids, named after their generally yellow color in nature (Lat. *flavus* = yellow) are a class of phenolic secondary metabolites produced by plants and fungi, containing now over 15,000 naturally occurring compounds [1]. Their general structure consists of a 15-carbon skeleton, forming two phenyl (A and B) and one heterocyclic ring (C), abbreviated C6-C3-C6 [2]. They comprise (i) flavonoids, derived from 2-phenyl-chromen-4-one (2-phenyl-1,4-benzopyrone); (ii) isoflavonoids, having the 3-phenyl-chromen-4-one structure (e.g., genistein, daidzein) and (iii) neoflavonoids, derived from 4-phenyl-coumarine (4-phenyl-1,2-benzopyrone). The first group is further divided into flavones (luteolin, apigenin), flavonols (quercetin, kaempferol, myricetin), flavanones (hesperetin, naringenin) and flavanonols (taxifolin). Moreover, the term flavonoid often also describes non-ketone containing polyphenols, such as flavanoids, e.g., flavan-3-ols (catechin, epicatechin, theaflavin and related compounds) or anthocyanidins, containing the flavylium (2-phenylchromenylium) ion backbone (e.g., cyanidin) [2,3]. Flavonoid structures are in nature often further derivatized, inter alia, prenylated as in the case of icaritin [4] (8-prenylkaempferide, Figure 1).

Flavonoids are probably the most common polyphenols in the human diet, ubiquitous in the plant kingdom. In plants, they provide color to flowers and fruit and protect them against herbivores and from the excess of solar ultraviolet (UV) radiation [5]. Flavonoids are widely distributed in nature, usually in the form of various glycosides, such as glucosides, galactosides, rhamnosides, arabinosides and rutinosides [6]. Since diets rich in flavonoids have repeatedly been related to low incidence of cardiovascular, neurodegenerative, and oncological diseases [3], various food supplements containing these compounds are becoming increasingly popular among the general population. Some flavonoid glycosides can quite easily be isolated from natural sources. Others, in contrast, are present in natural material in so low amounts that their isolation is rather unrealizable. Modifications of the glycosidic parts of the abundant flavonoids—selective trimming on one side and glycosylation on the other—are obviously an effective means for the production of their derivatives.

Glycosylation of flavonoid compounds is considered an efficient approach to increase their solubility and stability in water [7], which also leads to improvement of the pharmacological properties of flavonoids by increasing their bioavailability and sometimes decreasing acute toxicity or harmful effects [8]. In nature, the glycosides of phenolic compounds are typically synthesized by regio- and stereospecific glycosyltransferases [9]; these enzymes are often used in biotechnology for the synthesis of a variety of glycosides of phenolics [10,11] and other bioactive compounds [12]. However, the major obstacle in the widespread use of glycosyltransferases is their poor stability and generally expensive glycosyl donors, typically sugar nucleotides, which brings along the necessity of a donor regeneration system for larger productions [13]. This may be overcome by whole-cell biotransformation setup, where the cells producing the glycosyltransferase activity can provide the required recycling of the costly donors in situ [14,15]. In the past decade, both natural and engineered glycosidases [16] have come into play as some of them have been shown to possess the ability of transferring glycosyl moieties onto phenolic hydroxyls [17,18,19]. They offer robustness, stability, scalable production, affordable substrates and a simple reaction design without the need of in situ regeneration of nucleotide sugar substrates [20]. Moreover, the natural hydrolytic activity of glycosidases is widely employed in biotechnological deglycosylation processes producing respective aglycones or partially deglycosylated flavonoids [21,22,23]. Deglycosylation is quite commonly used in the food industry aiming at the improvement of sensoric properties of beverages such as debittering of citrus juices [24] or enhancement of wine aromas [21].

The study of flavonoid glycosides borders on two scientific disciplines: polyphenol and carbohydrate research. The researchers focusing on polyphenols usually have troubles to master the carbohydrate chemistry and do often not recognize the nuances sprouting from the complicated nomenclature of the glycosidic parts of flavonoids. Typically, the glycosidic part is described only as e.g., rha-glc instead of 2-*O*-β-d-glucopyranosyl-α-l-rhamnopyranosyl neglecting the absolute configuration at the anomeric carbon or the position of the glycosidic bond [4]. On the other hand, glycoscientists might not clearly distinguish various molecules of flavonoids, the most common problems arise from similarities in the trivial names of e.g., quercetin, quercitrin, isoquercitrin and isoquercetin or hesperidin and hesperetin. This sometimes results in the imprecise chemical structures of flavonoid glycosides and various inconsistencies found in scientific reports [4,13,25]. The present review is based on complementary expertise in both fields and focuses on recent advances in the modifications of the glycosidic parts of various flavonoids using glycosidases, covering both selective trimming of the sugar moieties and glycosylation of flavonoid aglycones by defined natural and mutant glycosidases. The differences in the properties and physiological effects of the glycosylated and deglycosylated flavonoids are also discussed. Biotransformations of flavonoids with glycosyltransferases and undefined microbial enzymes [6,8] are not included in the present work.

## 2. Glycosidase-Catalyzed (de)Glycosylations

Glycoside hydrolases (EC 3.2.1.-), often also referred to as glycosidases, are enzymes catalyzing the cleavage of terminal (*exo*-) or internal (*endo*-enzymes) *O*-, *N*- and *S*-glycosidic bonds in carbohydrate chains and various glycoconjugates. Besides the International Union of Biochemistry and Molecular Biology (IUBMB) Enzyme Nomenclature classification based on substrate specificity, the Carbohydrate-Active Enzymes database (CAZy; available online: http://www.cazy.org) has divided glycosidases into 153 glycoside hydrolase (GH) families based on amino acid sequence and thus structural similarities of the enzymes so far [26].

**Scheme 1:**
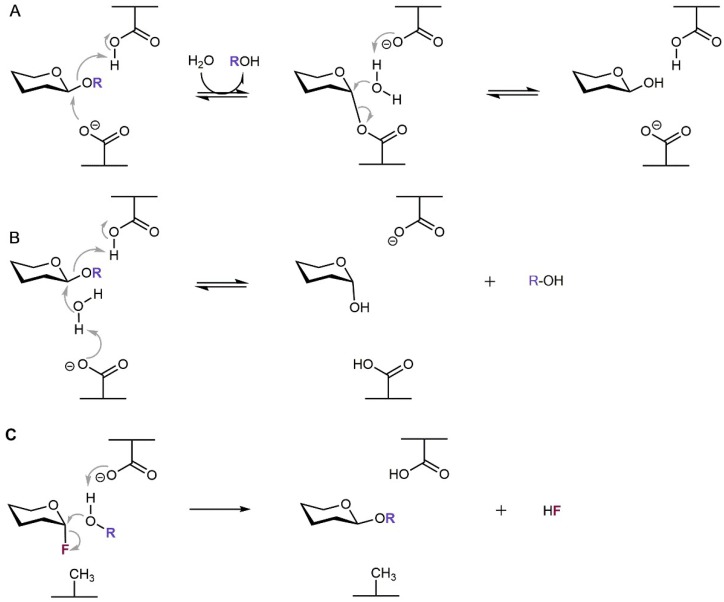
Catalytic mechanisms of glycosidases. (**A**) Retaining β-glycosidase. (**B**) Inverting β-glycosidase. (**C**) Glycosynthase originated from a retaining β-glycosidase. The aglycone and fluor atom are highlighted by violet and dark red, respectively.

Generally, two different catalytic mechanisms for the retaining and inverting modes of action have been proposed for glycosidases by Koshland as early as in 1953 [27]; since then they have been revised and described in more detail based on current advances in enzyme structure knowledge [28]. In most glycosidases the catalytic amino acid residues are represented by a pair of two carboxylic residues of aspartate and/or glutamate. Retaining glycosidases proceed via the two-step double-displacement mechanism, in which the covalent glycosyl-enzyme intermediate is formed, while they retain the stereochemistry at the substrate’s carbohydrate anomeric center. Here, the mechanism involves a general acid/base catalytic residue that facilitates the leaving group departure in the first step while the other residue stabilizes the reaction intermediate usually by forming a covalent glycosyl-enzyme intermediate. In the consequent step the acid/base residue facilitates the attack of the water molecule (or other acceptor in case of transglycosylation) on the anomeric center to degrade the intermediate while forming the product with preserved stereochemistry (Scheme 1A). On the other hand, glycosidases operating with inversion of stereochemistry at the substrate’s anomeric center employ concomitant general acid catalysis to enable the departure of the leaving group and general base catalysis facilitating the attack of water molecule at the anomeric center (Scheme 1B). 

**Scheme 2:**
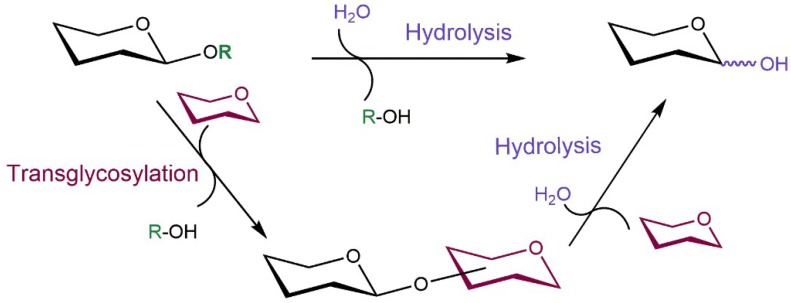
Hydrolytic and transglycosylation reactions performed by glycosidases. The aglycone, transferred sugar moiety and water (and related processes) are highlighted in green, dark red and violet, respectively.

From their definition, the natural function of glycosidases is the hydrolysis of glycosidic bonds. However, upon reaction engineering, comprising concentrations of carbohydrate substrates and their suitable activation, they may be employed for the synthetic aims because, contrary to glycosyltransferases, they are usually stable and robust and the glycosyl donors employed in glycosidase-catalyzed reactions are affordable. Transglycosylation is a kinetically controlled process (under favorable kinetic conditions concentrations of products higher than the concentrations corresponding to reaction equilibrium can be reached), catalyzed typically by the retaining glycosidases, in which the glycosyl moiety is transferred from the activated glycosyl donor onto an acceptor bearing a free hydroxyl group instead of water (Scheme 2). With wild-type glycosidases the synthetic yields hardly exceed 40% due to the concurrent hydrolysis of both the substrates and the transglycosylation products [29], though there are exceptions [30]. This drawback has been removed in glycosynthases, the pioneers of engineered glycosidases [31,32,33]. By replacing the active-site catalytic nucleophile by a small neutral amino acid residue, glycosynthases lose their hydrolytic activity and process only special substrates-glycosyl fluorides in the opposite anomeric configuration to the natural substrate, which they transfer to a variety of acceptors, creating a glycosidic linkage (Scheme 1C). A large number of glycosyl fluorides employed as glycosynthase substrates have been prepared so far, they are easily synthesized and fairly stable [34]. Moreover, transglycosidases strongly preferring transglycosylation over hydrolysis and accepting substrates of their natural configuration, have been reported both wild-type [19] and engineered from retaining glycosidases [35]. An additional advantage of glycosidases is their broad tolerance toward structural modifications in their substrates, which enabled glycosylations with modified carbohydrates [36,37,38].

A special group of enzymes is constituted by glycoside phosphorylases that, besides their hydrolytic activity toward sugar-1-phosphates, feature a glycosyltransferase-like activity using simple donors such as glycosyl phosphates and sucrose. In the CAZy system based on sequence and structure similarities they belong to several GH families (e.g., sucrose phosphorylase belongs to GH13) [39]. Even though these enzymes are still quite rare, they have already shown their utility in glycosylation of phenolic compounds including flavonoids [17]. Therefore, glycosyl phosphorylase-catalyzed glycosylations will also be discussed farther in this paper.

## 3. Differences in Biophysical, Biochemical and Physiological Properties of Glycosylated and Deglycosylated Forms of Flavonoids

Glycosylation naturally affects biophysical and biochemical properties of flavonoids, as well as their biological activities [1,7].

### 3.1. Solubility, Lipophilicity/Hydrophilicity and Stability

One of the important parameters affected by the presence of glycosyl moieties in the structure of flavonoids is their solubility in both aqueous and organic solvents. Flavonoids are usually poorly soluble in aqueous solutions and tend to spontaneously oxidize there forming insoluble polymers [40]. As flavonoids are weak acids due to presence of phenolic groups, their water solubility is enhanced in alkaline pH [22], but their dietary sources are often acidic (fruit) and pH is changing also in human gastrointestinal tract. While the solubility of flavonoids in lipophilic solvents partially prevents them from oxidation in food sources, their solubility in water generally improves their bioavailability from the diet [7] and glycosylation usually increases aqueous solubility [7]. Thus, aqueous solubility of myricitrin was increased 480 times by its galactosylation [41] and that of puerarin was enhanced 14 and 168 times by its glucosylation and maltosylation, respectively [42]. However, the effect is dependent on the respective sugar moieties. While glucosylation or galactosylation usually increases water solubility, the presence of rhamnosyl residues in rutinosides or neohesperidosides slightly decrease it. For instance, solubility of quercetin and its glycosides increased in the order rutin < quercetin < isoquercitrin < enzymatically modified isoquercitrin (EMIQ) [43]. Similarly, in the case of hesperetin, the most soluble in both water and 10% ethanol was hesperetin-7-*O*-glucoside, followed by the rutinoside hesperidin and the aglycone was the least soluble [44]. On the other hand, glycosylated flavonoids such as isoquercitrin and rutin are less soluble than their aglycone in polar organic solvents [45,46].

Solubility in aqueous and organic solvents is related to hydrophilic/lipophilic properties of glycosylated flavonoids, which then determine e.g., their interphase partitioning, protein and membrane interactions, transport and binding activity, in vivo bioavailability, absorption, excretion and the biological activity. Hydrophilicity and lipophilicity (hydrophobicity) are usually assessed using the octanol-water (buffer) partition coefficient *P* defined as a particular ratio of the concentrations of a solute between the two solvents (the lipophilic phase in the numerator and hydrophilic phase in the denominator) and expressed as log *P*. This parameter is strongly affected by glycosylation of flavonoids, which enhance their hydrophilic properties and therefore decrease log *P*. Accordingly, log *P*_oct/wat_ of quercetin and its glycosides decreased as follows: quercetin > isoquercitrin > EMIQ > rutin [47,48]. The position and number of the sugar moieties also play important roles, as exemplified by the partition coefficient of quercetin glucosides decreasing in the order: 4′-*O*-glucoside > 3-*O*-glucoside > 3,4′-*O*-diglucoside [47,49].

Glycosylation affects also the stability of flavonoid molecules toward oxidative degradation, such as boiling at 100 °C in the presence of air, metal- or peroxidase-catalyzed oxidative degradation, usually by enhancing it as in the case of rutin compared to quercetin or quercetin-4′-glucoside compared to quercetin-3,4′-diglucoside [7]. Glycosylation often improves the stability of the polyphenols by mere blocking of phenolic group(s), which could be apt to oxidation or radical attack. This is also linked with fact that glycosylated flavonoids often exhibit lower antioxidant/antiradical capacity mainly in the in vitro tests (such as 1,1-diphenyl-2-picrylhydrazyl (DPPH) or 2,2′-azinobis-(3-ethylbenzothiazoline-6-sulfonic acid) (ABTS) radical scavenging, etc.) caused by lower number free phenolic groups available (see Section 3.3).

### 3.2. Bioavailability

Bioavailability of glycosylated flavonoids is usually assessed as the concentration of the (conjugated) aglycone in blood serum or plasma, because the glycosidic parts are usually thought to be cleaved before absorption. In a certain sense, *O*-glycosides serve as “pro-drugs” thanks to dramatically enhanced solubility of aglycones via their binding to sugar moieties [1]. The presence of sugar moieties usually leads to enhanced bioavailability of the respective flavonoid aglycone depending on the nature of the sugar as e.g., glucosides are absorbed more rapidly than other types of glycosides such as rhamnosides and rhamnoglucosides, i.e., rutinosides or neohesperidosides [50]. This is mainly due to the presence/absence of respective hydrolyzing enzymes in the human gastrointestinal tract. While glucosides can be cleaved by intestinal lactase-phlorizin hydrolase or small intestinal epithelial cell β-glucosidase, no human α-l-rhamnosidase or rutinosidase exists and the bioavailability of rhamnose containing flavonoids is fully dependent on their cleavage by intestinal microbiota [40,51]. Moreover, not all intestinal strains have this ability [52] and in some subjects, such strains seem to be (almost) completely absent. This contributes to large inter-individual variability in the bioavailable level of the respective aglycones [53]. Indeed, quercetin from isoquercitrin was shown to be better absorbed than quercetin, rutin and quercitrin in rats [54] and pigs [55] and similar to that of spiraeoside in healthy humans [56]. The reason dwells probably in the dramatically different structure of rhamnose compared to other sugars usually present in flavonoid glycosides. Not only it is a deoxy sugar (6-deoxy-l-mannose), but it naturally occurs, in contrast to most natural sugars in its l-form. Unfortunately, almost no data on the bioavailability of other known l-glycosides, such as guaijaverin or avicularin (Figure 1) are available. Interestingly, several recent studies (all by the same laboratory) found considerable amounts of intact glycosides such as hyperin, reynoutrin, guaijaverin, rutin, quercitrin, isoquercitrin or astragalin shortly (within minutes) after their administration in rat and mice plasma by liquid chromatography–tandem mass spectrometry (LC-MS/MS) [57,58,59,60]. When the aglycone was measured simultaneously, it displayed a concentration maximum within minutes after administration as well, but also a second broad peak at 6–8 h indicating plausible cleavage of glycosides and/or enterohepatic recirculation of the aglycone [57]. Unfortunately, conjugated metabolites were not determined in these studies. Potential presence of intact glycosides is often masked by enzymatic or acidic hydrolysis of plasma samples routinely used in pharmacokinetic studies, which leads also to the cleavage of glycosidic bonds [40]. The mechanism of the absorption of intact glycosides was thought to involve Na^+^-dependent glucose transporter 1 (SGLT1) on epithelial cells [61], but this has been found to not be the case [62] and more studies are needed to elucidate this phenomenon.

### 3.3. Biological Activity

The impact of glycosylation on the biological activity of flavonoids have been reviewed recently [45,63] and therefore, this aspect will be only briefly summed up and illustrated by several examples here. Glycosylated flavonoids generally display lower antioxidant capacity compared to the corresponding aglycones, the activity usually decreases with increasing number of glycosidic moieties and the position and structure of the sugar(s) play important roles [1,7,64,65]. This is in accordance with the structural elements of flavonoids crucial for their radical scavenging activity: the *o*-dihydroxy (catechol) structure in the B-ring; the C-2,C-3-double bond in conjugation with the 4-oxo group in the C-ring and the presence of hydroxyl groups in positions C-3 (C-ring), C-5 and C-7 (A-ring) [40,65]. In quercetin bond dissociation enthalpies for the OH-groups increase in the order C-4′-OH < C-3′-OH < C-3-OH < C-7-OH < C-5-OH [66]. Once the respective OH-group is glycosylated, it becomes logically unusable for hydrogen atom abstraction necessary for radical scavenging action of flavonoids. Glycosylation seems to generally reduce not only antioxidant activity, but also antidiabetic, anti-inflammatory, antibacterial, antifungal, antitumor, anticoagulant, antiplatelet, antidegranulating, antitrypanosomal, immunomodulatory, and anti-tubercular activity, influenza virus neuraminidase and aldehyde oxidase inhibition [1]. On the other hand, glycosylation can enhance certain types of biological benefits including anti-human immunodeficiency virus (HIV), anti-rotavirus, anti-stress, anti-obesity, anti-adipogenic and anti-allergic activity, tyrosinase inhibition or anticholinesterase potential [1].

Here again, the specific properties of rhamnose seem to play an important role, as isoquercitrin displayed higher scavenging and antiproliferative activities than rutin [67]. Similarly, hesperetin-7-*O*-glucoside was better than both hesperetin and hesperidin in inhibiting human intestinal maltase. In the case of human 3-hydroxy-3-methyl-glutaryl-coenzyme A (HMG-CoA) reductase inhibition, the activity increased in the order: hesperidin < hesperetin-7-*O*-glucoside ≈ hesperetin [44]. On the other hand, hesperidin and neohesperidin showed considerably higher anti-proliferative effects than hesperetin and rutin on human hepatoma (HepG2) cells, but lower DPPH scavenging activity [63]. In the case of prenylated flavonoids from *Epimedium koreanum*, i.e., icariin, icaritin, icariside II, epimedin A, epimedin B, and epimedin C (for structures, see Figure 1), only icaritin and icariside II, both lacking the glucose moiety at C-7, were able to inhibit protein tyrosine phosphatase 1B and α-glucosidase with an antidiabetic potential [4].

## 4. Selective Hydrolysis of Glycosylated Flavonoids

**Scheme 3:**
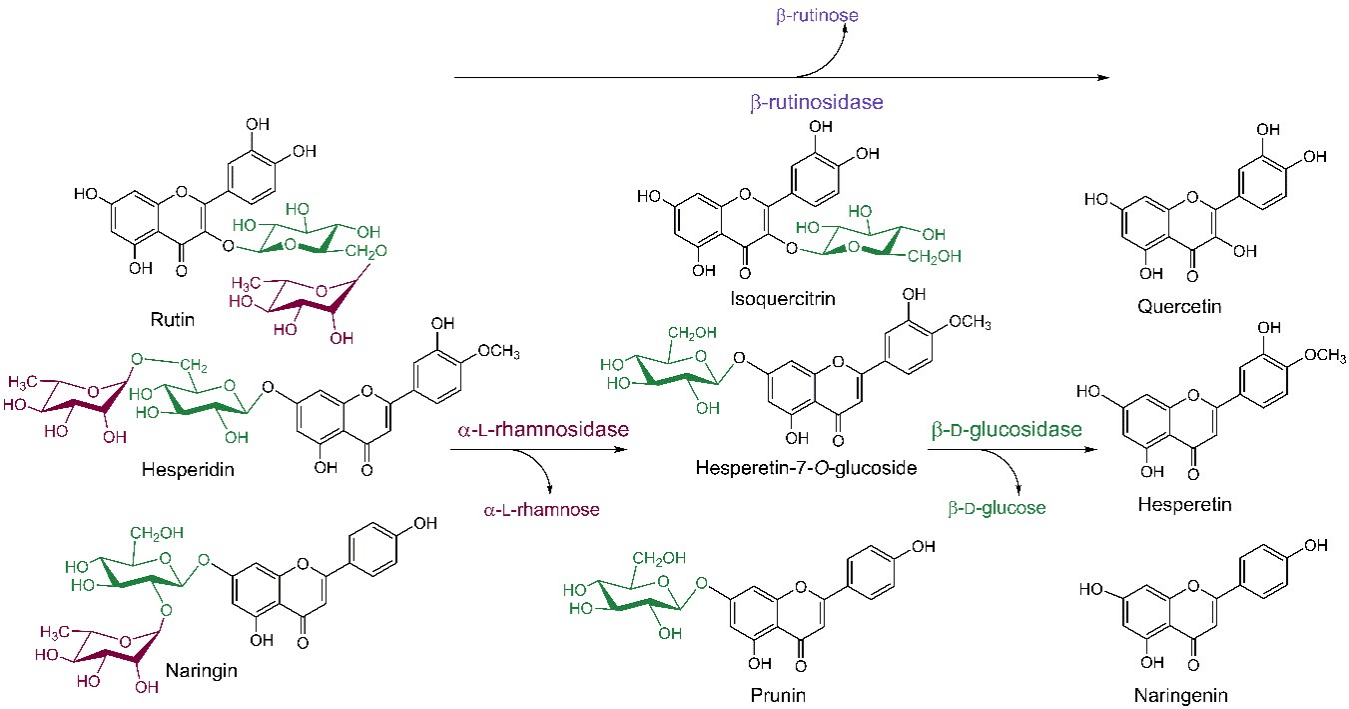
Deglycosylation reactions of rutin, hesperidin and naringin catalyzed by glycosidases and their respective products. β-d-Glucopyranose, α-l-rhamnopyranose and β-rutinose (and related enzymes) are highlighted by green, dark red, and violet colors, respectively.

Flavonoids are abundant metabolites of vascular plants and therefore a regular part of human diet with many beneficial effects on human health (see Section 3). Rutin, hesperidin and naringin belong to the most widespread storage forms of glycosylated flavonoids found mainly in buckwheat, apple, grape, tomato and citrus fruit [68,69,70]. These compounds contain the α-l-rhamnosyl-β-d-glucoside disaccharide moiety bound to the flavonoid aglycone at the position 3 (rutin) or 7 (hesperidin, naringin). The glycosidic linkage between the two sugar units is either β1→2 (disaccharide neohesperidose) or β1→6 (disaccharide rutinose), which brings along the need to use selective glycosidases for the hydrolysis of the glycosidic bonds both within the disaccharide and between the carbohydrate moiety and the flavonoid aglycone (Scheme 3). Rutin and hesperidin are easily isolated from the agro- and fruit juice-producing industries in large amounts and high quality for affordable prices, which makes them a perfect starting material for the preparation of the more valuable flavonoid glucosides (mainly isoquercitrin) and free flavonoids. Naringin is an undesired component of citrus juices causing their natural bitterness, while the deglycosylated naringenin is tasteless. Thus, citrus juice industry largely employs glycosidases to improve sensoric qualities of their products. Advantageously, the α-l-rhamnosidase-catalyzed hydrolysis yields substantial amounts of free α-l-rhamnose as a by-product, which is used in the cosmetic industry in the form of rhamnolipids as an anti-wrinkle agent [71]. Rutinose, the disaccharide produced by the action of rutinosidase on rutin and hesperidin, has not been produced in sufficient amounts to test its properties so far due to the lack of available rutinosidases; however, there is a potential that needs to be explored.

For the enzymatic hydrolysis of the above flavonoid glycosides, three principal glycosidases are used: (i) α-l-rhamnosidases (EC 3.2.1.40), which cleave off the α-l-rhamnosyl moieties; (ii) β-d-glucosidases (EC 3.2.1.21), hydrolyzing the glycosidic bond between the glucose moiety and the flavonoid aglycone; and (iii) β-rutinosidases (EC 3.2.1.168), which are rare diglycosidases cleaving off the entire disaccharide rutinose from the aglycone (Scheme 3). Often the term naringinase is found in the literature, which stands for the complex of α-l-rhamnosidase with β-d-glucosidase produced by many microorganisms and is widely employed particularly in the citrus juice debittering [24]. 

A vast majority of the reported α-l-rhamnosidases have been produced by filamentous fungi, namely the *Aspergillus* and *Penicillium* genera [22,72,73,74,75]; however, bacterial α-l-rhamnosidases have been studied as well [76,77,78,79,80,81]. To facilitate the large scale production of these enzymes, some of the microbial α-l-rhamnosidases have been recombinantly produced in *Pichia pastoris* expression system [82,83,84]; moreover, immobilization on a variety of carriers is a frequent way to increase the stability and enable recycling of the produced enzymes [79,85,86]. α-l-Rhamnosidases isolated from various producers differ mainly in their substrate specificities as some of them are promiscuous and hydrolyze the terminal α-l-rhamnosyl unit from a large array of substrates [72,87,88] while some enzymes feature a strong specificity toward one substrate, e.g., rutin [75] and hesperidin [89]. Typically, β-d-glucosidases are not used as separate enzymes, but they are present in the commercial naringinase and hesperidinase complexes employed in the industrial deglycosylation of naringin. As an example of the rare use of pure β-d-glucosidases, this enzyme was utilized in the selective hydrolysis of naturally occurring icariin to afford the more valuable icariside II (Figure 1) with high purity [90]. For the specific production of the valuable prunin and isoquercitrin starting from naringin and rutin, respectively, the β-d-glucosidase activity needs to be suppressed to avoid complete deglycosylation of the flavonoids. This can be achieved by selection of appropriate pH and temperature conditions unfavorable for the β-d-glucosidase activity while the α-l-rhamnosidase activity is retained [23,91]. Unfortunately, this is not always the case and some authors neglect the presence of β-d-glucosidase in naringinase preparations [25].

To reach higher productivity of the α-l-rhamnosidase-catalyzed deglycosylations, several atypical reaction setups have been explored. The productivity and conversion of the hydrolytic reactions were significantly increased by physical effects such as ultrasound irradiation [92] and high hydrostatic pressure [93]. As the water solubilities of both the substrates and products are rather poor, the hydrolytic reactions with glycosidases in co-solvent systems as well as in an ionic liquid-buffer biphase system have been optimized [94,95]. An ingenious way of immobilization of α-l-rhamnosidase was reported by Liu and co-workers, who expressed a fungal α-l-rhamnosidase on the cell surface of *Saccharomyces cerevisiae* and successfully employed the yeast cells as a catalyst for debiterring of citrus juices with the α-l-rhamnosidase activity retained for ten batches [88].

Typically, enzymatic hydrolysis of glycosylated flavonoids is performed in low substrate concentrations (mM), due to their poor solubility, which hampers higher productivity of the reactions [25]. To increase the yields of the valuable product, Křen and co-workers developed a method for the preparatory production of isoquercitrin from rutin employing a thermostable and alkali-tolerant α-l-rhamnosidase from *Aspergillus terreus*. This enzyme, selected in an extensive multifactorial screening, enables to operate at 70 °C and pH 8.0 with a high substrate load of up to 300 g/L (in suspension). Moreover, the reaction has been scaled-up to 10 kg production, yielding pure isoquercitrin (98%) not contaminated by quercetin, outright usable in nutraceuticals [22,96]. To support the biotechnological potential of the α-l-rhamnosidase from *A. terreus*, the recombinant enzyme was immobilized in polyvinylalcohol hydrogel with excellent storage stability and no apparent loss of activity after 27 reaction runs [86]. The recombinant production of this enzyme in *Pichia pastoris* was optimized and scaled-up in a 10-liter working volume fermenter [84]. Additionally, the detailed study of the α-l-rhamnosidase from *A. terreus* revealed its unique ability to synthesize a variety of α-l-rhamnosides including acceptors with phenolic hydroxyls such as hydroquinone and catechol by reverse hydrolysis reaction [97].

Rutinosidases (6-*O*-α-l-rhamnopyranosyl-β-d-glucopyranosidases) form a small group of microbial retaining diglycosidases able to cleave off the entire diglycoside rutinose from a variety of substrates. Rutinosidases, as a quite new group of enzymes, have recently become of scientific interest as they usually exhibit significant transglycosylation activity [19,98]. Typically, rutinosidases are specific to the 3- or 7-*O*-position of the sugar moiety at the flavonoid molecule, thus preferably hydrolyzing either rutin [99] or hesperidin [100]. The hesperidin-specific rutinosidase from *Acremonium* sp. was immobilized on several chitosan and agarose-based supports to stabilize the enzyme against elevated temperature and high concentration of dimethyl sulfoxide, enabling to reutilize the enzyme for up to 18 cycles of hesperetin production [101,102]. This enzyme was also employed in the development of the enzymatic-spectrophotometric method for quantification of hesperidin in raw orange juices by the hydrolysis of hesperidin to hesperetin and measuring the product’s absorbance in the UV range at 323 nm without the need of flavonoid extraction [103]. Interestingly, the rutinosidase isolated from the hyperthermophile *Pyrococcus furiosus* was able to deglycosylate rutin to quercetin at 95 °C with a half-life of 101 h [104].

## 5. Glycosylation of Flavonoids by Glycosidases 

**Scheme 4:**
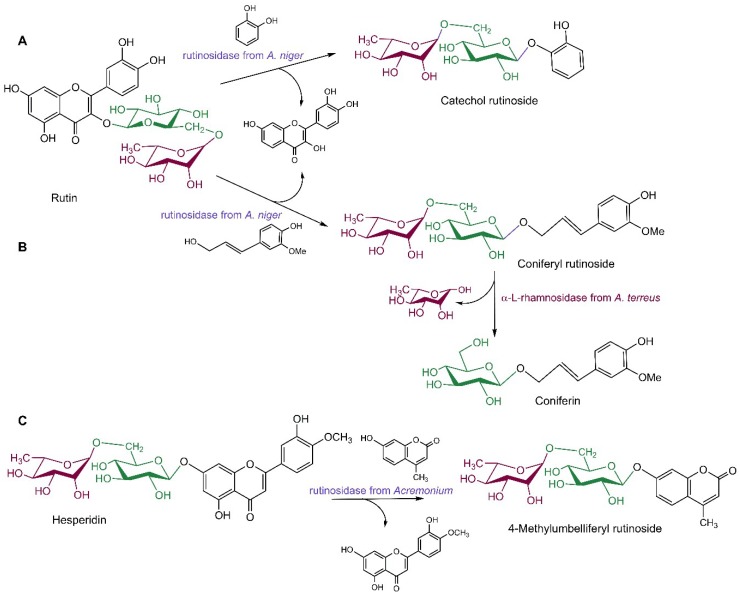
Rutinosidase-catalyzed transglycosylations. (**A**) Transglycosylation reaction using rutin as glycosyl donor and catechol as an acceptor catalyzed by rutinosidase from *Aspergillus niger* [19]. (**B**) Transglycosylation reaction using rutin as glycosyl donor and coniferyl alcohol as an acceptor catalyzed by rutinosidase from *A. niger*; the transglycosylation product is subsequently cleaved by α-l-rhamnosidase from *A. terreus* to yield coniferin [106]. (**C**) Transglycosylation reaction using hesperidin as glycosyl donor and 4-methylumbelliferone as an acceptor catalyzed by rutinosidase from *Acremonium* sp. [103]. β-d-Glucopyranose, α-l-rhamnopyranose and β-rutinose (and related enzymes) are highlighted by green, dark red, and violet colors, respectively.

Glycosidases have recently become a viable means of glycosylation of a variety of compounds due to their natural or engineered transglycosylation activity [16,105]. Transglycosylation is a process, in which the glycosyl moiety is transferred from an activated donor to an acceptor bearing a free hydroxyl group other than water. The optimum reaction conditions for transglycosylations must be finely tuned to maximize the yields of the desired product and suppress the concurrent hydrolytic activity of the glycosidase used. There are three types of transglycosylation reactions where flavonoids are involved: (i) glycosylated flavonoid is used as a glycosyl donor; (ii) glycosylation of the free hydroxyl groups of flavonoid aglycones; and (iii) transglycosylation in which the sugar moiety of a glycosylated flavonoid acts as an acceptor (Scheme 4). All these reaction modes are presented further in this section.

Rutinosidases (in the literature also termed α-rhamnosyl-β-glucosidases) have shown their universal utility in transferring the rutinosyl moiety to a wide array of aliphatic and aromatic hydroxyls, the latter being a rare capability among glycosidases. Using the rutin-specific rutinosidase from *Aspergillus niger* and rutin as glycosyl donor, several new aliphatic and aromatic rutinosides have been prepared reaching yields up to 46% (Scheme 4A) [19]. In another study, with this enzyme a series of arylakyl alcohols were first rutinosylated, subsequently the disaccharide was cleaved by α-l-rhamnosidase from *Aspergillus terreus* to obtain the desired bioactive glucosides (Scheme 4B) [106]. Also, the hesperidin-specific rutinosidase from *Acremonium* sp. exhibited a broad spectrum of rutinosyl acceptors, enabling the synthesis of wine aroma precursors which can be employed as standards in food analysis [107]. Moreover, using the immobilized *Acremonium* rutinosidase, the fluorogenic substrate of rutinosidases, 4-methylubelliferylrutinoside, was prepared with 16% yield (Scheme 4C) [18].

**Scheme 5:**
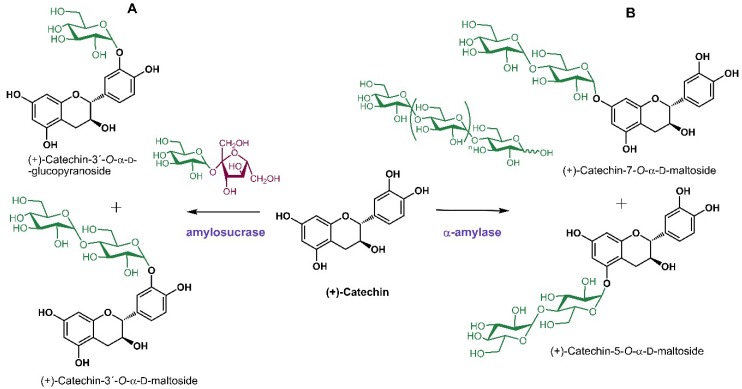
Enzymatic glycosylations of (+)-catechin. (**A**) Transglycosylation reaction using sucrose as donor catalyzed by amylosucrase from *Deinococcus geothermalis* [108]. (**B**) Transglycosylation reaction using dextrin as donor catalyzed by α-amylase from *Bacillus* sp. [110]. α-d-Glucopyranose, β-d-fructofuranose and glycosylating enzymes are highlighted by green, dark red, and violet colors, respectively.

Catechins are the basic structural units of condensed tannins featuring many favorable physiological activities, which are poorly bioavailable due to their low solubility in aqueous media. (+)-Catechin has been used as a model compound for glycosylation reactions as it is a structurally representative flavonoid compound available at a reasonable cost. Several different enzymes were employed in the glycosylation of (+)-catechin; interestingly, 3′-glucosides and maltosides were obtained when phosphorylases were used as catalysts [108,109] while 5, 7 and 4′-glycosides stemmed from the reactions catalyzed by classical glycosidases such as cellulase and α-amylase [110] (Scheme 5). Commercial cellulase from *Penicillium decumbens* was successfully utilized in the glucosylation of flavonoids abundant in the leaves of *Ginkgo biloba* (quercetin, kaempferol and isorhamnetin) using maltose as glucosyl donor. The discrepancy in the substrate configuration (cellulase is specific for the cleavage of β-glycosidic bonds, while maltose is constituted of two α-linked glucose units) suggests that the cellulase preparation also comprises α-specific glucosidases. The increased solubility of the resulting flavonoid glucosides facilitated their efficient extraction from the plant material [111].

Mutant glycosidases able to transfer glycosyl moieties directly onto the flavonoid hydroxyl group have also been reported. The glycosynthase E197S mutant of the Cel7B glycosidase from *Humicola insolens* exhibited transfer of the lactosyl moiety from lactosyl fluoride to e.g., quercetin reaching reaction rates comparable to those of uridine diphosphate (UDP)-dependent glycosyltransferases with the same receptors [112]. The glycosynthase mutants of β-glucosidase from the thermophilic bacterium *Thermotoga neapolitana* were employed in the glycosylation of isoquercitrin, yielding quercetin-3,4′-diglucoside with poor yields, suggesting that flavonoids are not the acceptors preferred by these enzymes [113]. Recently, the R134A mutant of sucrose phosphorylase from *Thermoanaerobacterium thermosaccharolyticum* was identified as an efficient catalyst to produce quercetin and catechin glucosides using simple sucrose as glucosyl donor [17].

**Figure 2:**
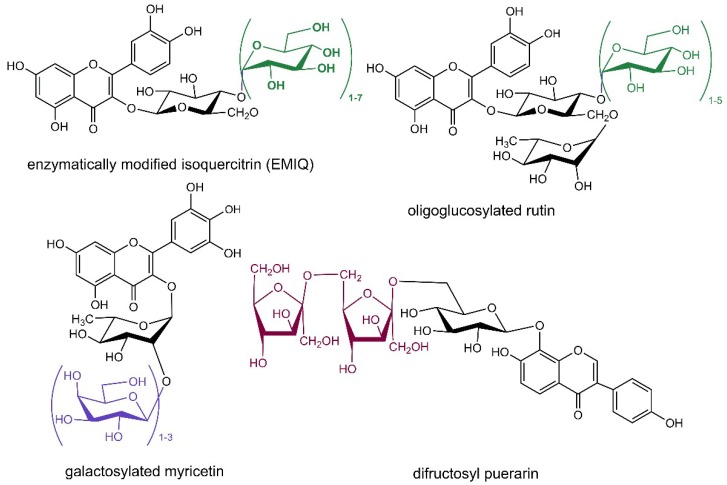
Structures of oligoglycosylated conjugates of flavonoids obtained by transglycosylation reactions. The conjugated sugar moieties are shown in green (β-d-glucopyranose), violet (β-d-galactopyranose) and dark red (β-d-fructofuranose) color.

In another approach, the oral bioavailability of favorably bioactive flavonoids is strongly enhanced by their conjugation with oligoglycosides, the typical acceptor of the glycosylation is the sugar moiety of the original flavonoid glycoside. The most elaborated example is the synthesis of EMIQ (Figure 2), which has gained the Generally Recognized as Safe (GRAS) notification from the American Food and Drug Administration as a mixture of compounds protecting flavors and colors in foods [114]. EMIQ is prepared from rutin employing α-l-rhamnosidase to cleave off rhamnose and the resulting isoquercitrin is then poly-α-glucosylated using dextrin as glucosyl donor in the reaction catalyzed by cyclodextrin glucanotransferase [114]. When untreated rutin was used as glucosyl acceptor with this latter enzyme, a mixture of oligoglucosylated rutins (Figure 2) was acquired in the reaction accelerated by microwave irradiation in a short time with high efficiency [115]. Myricitrin was successfully galactosylated (Figure 2) employing β-galactosidase from *Bacillus circulans* with lactose as a sugar donor [41]. Puerarin, the 8-*C*-glucopyranoside of isoflavone daidzein naturally occurring in the Chinese herb *Radix puerarie* known for its beneficial effects in prevention and treatment of cardiovascular diseases and complications of diabetes mellitus, was glycosylated by an extremely thermostable maltogenic amylase utilizing β-cyclodextrin as glucosyl donor yielding a mixture of daidzein 8-*C*-glucosyl-(α-glucosyl)_1–6_ derivatives. Unfortunately, the obtained conjugates were not isolated; their structure was just elucidated from the high-performace liquid chromatography coupled with mass spectrometry (HPLC-MS) analysis [116]. On the other hand, a preparatory transglycosylation reaction was performed employing solvent tolerant β-fructosidase from *Arthrobacter nicotianae* and sucrose as the fructosyl donor reaching 90–99% yield of puerarin fructosides (Figure 2) without undesired hydrolysis of the products [117].

## 6. Conclusions

Natural and mutant glycosidases are an excellent tool for the modifications of the glycosidic parts of flavonoids through both selective trimming of the sugar moieties and glycosylation of flavonoid aglycones. The natural hydrolytic activity of glycosidases is widely employed in biotechnological deglycosylation processes producing respective aglycones or partially deglycosylated flavonoids. Moreover, deglycosylation is commonly used in the food industry for debittering of citrus juices or enhancement of wine aromas. On the other hand, glycosylation of flavonoids strongly enhances their water solubility and thus increases the bioavailability of these bioactive compounds. Antioxidant and most biological activities are usually suppressed in glycosides, but some specific bioactivities are enhanced. Importantly, the presence of l-rhamnose in rhamnosides, rutinosides and neohesperidosides seem to play an important role in the solubility, bioavailability and biological activity of the respective flavonoids. Overall, flavonoid glycosides may serve as “pro-drugs” that release the respective aglycones to the gastrointestinal tract.

## Abbreviations

Ara	α-l-Arabinopyranosyl
Ara*f*	α-l-Arabinofuranosyl
CAZy	Carbohydrate-Active Enzymes database
EC	Enzyme Commission number
EMIQ	Enzymatically modified isoquercitrin
Gal	β-d-Galactopyranosyl
GH	Glycoside hydrolase
Glc	β-d-Glucopyranosyl
GRAS	Generally Recognized as Safe
HMG-CoA	3-Hydroxy-3-methyl-glutaryl-coenzyme A
IUBMB	International Union of Biochemistry and Molecular Biology
Neo	Neohesperidosyl (2-*O*-α-l-rhamnopyranosyl-d-glucopyranosyl)
Rha	α-l-Rhamnopyranosyl (6-deoxy-α-l-mannopyranosyl)
Rha-Glc	2-*O*-β-d-Glucopyranosyl-α-l-rhamnopyranosyl
Rha-Rha	2-*O*-α-l-Rhamnopyranosyl-α-l-rhamnopyranosyl
Rha-Xyl	2-*O*-β-d-Xylopyranosyl-α-l-rhamnopyranosyl
Rut	Rutinosyl (6-*O*-α-l-rhamnopyranosyl-β-d-glucopyranosyl)
UV	Ultraviolet
